# Perspectives of family medicine residents in Riyadh on leadership training: a cross-sectional study

**DOI:** 10.1186/s12909-023-04188-2

**Published:** 2023-03-25

**Authors:** Yousef Alluhaymid, Abdulaziz Alalwan, Abdulmajeed Alruwaitea

**Affiliations:** Department of Family and Community Medicine, College of Medicine, University Family Medicine Center, King Saud University Medical City, Riyadh, Kingdom of Saudi Arabia

**Keywords:** Leadership, Family medicine, Residents, Saudi MED-FM

## Abstract

**Background:**

Medical educators in academia have faced challenges incorporating leadership training into curricula while minimizing redundancy and assuring value and relevance for all learners. This study aims to assess the status of leadership training as perceived by family medicine residents in Riyadh to advise the development of a formal leadership training curriculum.

**Method:**

The research is cross-sectional and quantitative. Participants were asked via an electronic questionnaire about their leadership attitudes, perceived degree of training in various leadership domains, and where they could find additional training.

**Results:**

The survey was completed by 270 family medicine residents in Riyadh. Residents rated the importance of physician leadership in their communities as high (6 out of 7 on a Likert scale). In contrast, agreement with the statement 'I am a leader' obtained the lowest grade (4.4 of 7 on a Likert scale). Overall, most of the residents participating in the study (50% or more) voiced a desire for more training in all leadership domains. Over 50% of residents indicated that leadership electives or selective lectures, workshops, or seminars as well as WADAs (Weekly Academic Day Activities), leadership mentors or coaches teaching junior learners (with training), and leadership courses could be incorporated into the curriculum to foster leadership skills.

**Conclusion:**

Residents were enthusiastic about family physicians being leaders, aligning with the current educational philosophy but requiring formal training. They also indicated areas where leadership training might be improved and developed in the current curriculum. This poll's results could be used to help residents build leadership skills by incorporating them into a formal leadership curriculum.

**Supplementary Information:**

The online version contains supplementary material available at 10.1186/s12909-023-04188-2.

## Introduction

As the recent coronavirus pandemic shows, health security is a critical aspect in developing countries and the world. Thus, many countries were eager to consider a substantial overhaul in the health care system.

Saudi Arabia's policy has heavily emphasized primary care and called on family physicians to assume various tasks, including leadership [1]. This is because ineffective leadership has been reported as the cause of Saudi Arabia's lack of a positive patient safety culture [2]. 

Medical organizations in Saudi Arabia have further supported these efforts by including leadership concepts and skills training in medical education frameworks that guide curriculum development, such as the Saudi Board for Family Medicine 2020 (SaudiMED-FM 2020) curriculum, in addition to other postgraduate programs of the Saudi Commission for Health Specialties [3]. The original Saudi Board for Family Medicine MED-FM curriculum was reviewed by the Saudi Commission for Health Specialties in 2019 to more accurately reflect what doctors are expected to do in today's health care system compared to the Saudi Board's previous edition [4].

Medical educators have difficulty incorporating leadership into curricula while minimizing redundancy and guaranteeing value and relevance for all learners. Coaching is touted as a teaching strategy that can help students move beyond rote memorization of facts to develop the process skills they'll need in the future. In recent years, there has been very little published in family medicine (FM) literature about these tasks [5, 6]. Postgraduate and graduate leadership education programs have remained relatively limited, according to numerous studies published between 1991 and 2017, which showed that the leadership curriculum is diversified and limited in effectiveness [7, 8].

As for undergraduate leadership training, there has been a consensus that this training should begin in medical school, but not on what it should look like or how it should be taught or evaluated [9].

The efficacy of an intervention depends in part on the target population's participation. Our study aims to assess the status of leadership training as perceived by family medicine residents in Riyadh to advise the development of a formal leadership training curriculum. This could address the lack of research on constructing formal leadership courses. The current project assessed how strongly residents associate family physicians with leadership, what domains of leadership residents desire more training in, and what opportunities residents identify to expand leadership training.

## Methods

### Setup, sampling, and process

Between January and April 2022, we conducted an observational, quantitative, cross-sectional study with FM residents in Riyadh City, Saudi Arabia. A self-administered questionnaire, previously used in another study, was given to participants to complete [6]. The questionnaire was handed out at different sites on different dates. The participants were asked to fill out the questionnaire only once to guarantee that no data was captured twice. King Saud University's College of Medicine Research Center in Riyadh, Saudi Arabia, gave its clearance for the study. There is a statement at the top saying, "Completion of this questionnaire will be treated as an indicator of your consent," as a way to get informed consent.

The questionnaire consisted of three parts. The first part assessed the resident’s agreement with leadership ideals. The second part assessed the exposure to leadership domains, and the final part identified leadership training opportunities during their residency. The LEADS framework, an evidence-based, comprehensive framework for health care leaders, includes a questionnaire for testing the following leadership dimensions [10, 11].

The inclusion criteria were all FM residents in Riyadh while the exclusion criteria were FM residents in the training centers outside Riyadh and all other Residents in other specialties inside or outside Riyadh. Using a single-proportion sample-size calculation, *n* = *z*^2^*p* (1–*p*)/*d*^2^, a 95% confidence level and a 5% margin of error were used to estimate the sample size. As a result, a sample size of 267 was required to estimate statistical significance. The questionnaire was distributed to all FM residents in all training centers during WADAs (Weekly Academic Day Activities) activity to give the same probability for each person to be included in the study.

Also, we tried to reduce recall bias by testing the recall period during the pilot study and allowing participants to contact one of the authors at times as follow-up or for any queries.

The survey underwent an extensive evaluation process in order to ensure its accuracy and relevance. In order to achieve this, a team of Five experts in the fields of Family Medicine and leadership were consulted. This team provided feedback on the language and content of the survey, and based on their suggestions, adjustments were made until a consensus was reached.

To assess the feasibility and interpretability of the survey, a trial run was conducted with seven recently graduated FM residents. The survey was designed to be completed in under 15 min and to maintain anonymity, it was paper-based and coded.

The leadership ideals of the participants were rated on a seven-point Likert scale, with 4 being considered neutral. Participants were also asked to identify their top three leadership opportunities. The results of the survey were analyzed using statistical methods, including counts, percentages, means, and standard deviations.

In order to determine the reliability of the leadership ideals section of the survey, a number of measures were used. The internal consistency of this section was evaluated using the Cronbach's alpha coefficient, which equaled 0.78. Additionally, the corrected item-total correlations ranged from 0.28 to 0.69, further pointing towards the satisfactory reliability of this section of the survey.

Finally, to determine any differences in leadership ideals across all resident responses and their perceived importance, a paired t test was used. The data analysis was performed using the Statistical Package for Social Studies (SPSS).

## Results

Overall, 270 residents participated in the study—134 of whom were female (49.6%). The mean age equaled 26.82, with a standard deviation of 2.63, a minimum of 18, and a maximum of 47. The participants were in their first (24.8%), second (26.3%), third (25.6%), and fourth (23.3%) year of residency. Most of the respondents did not have any leadership experiences longer than 20 h (77.0%) and did not participate in any leadership courses longer than 20 h (87.8%). The most common training places were Cluster 2 (21.1%) and King Khalid University Hospital (KKUH) (16.7%).

**Fig. 1:**
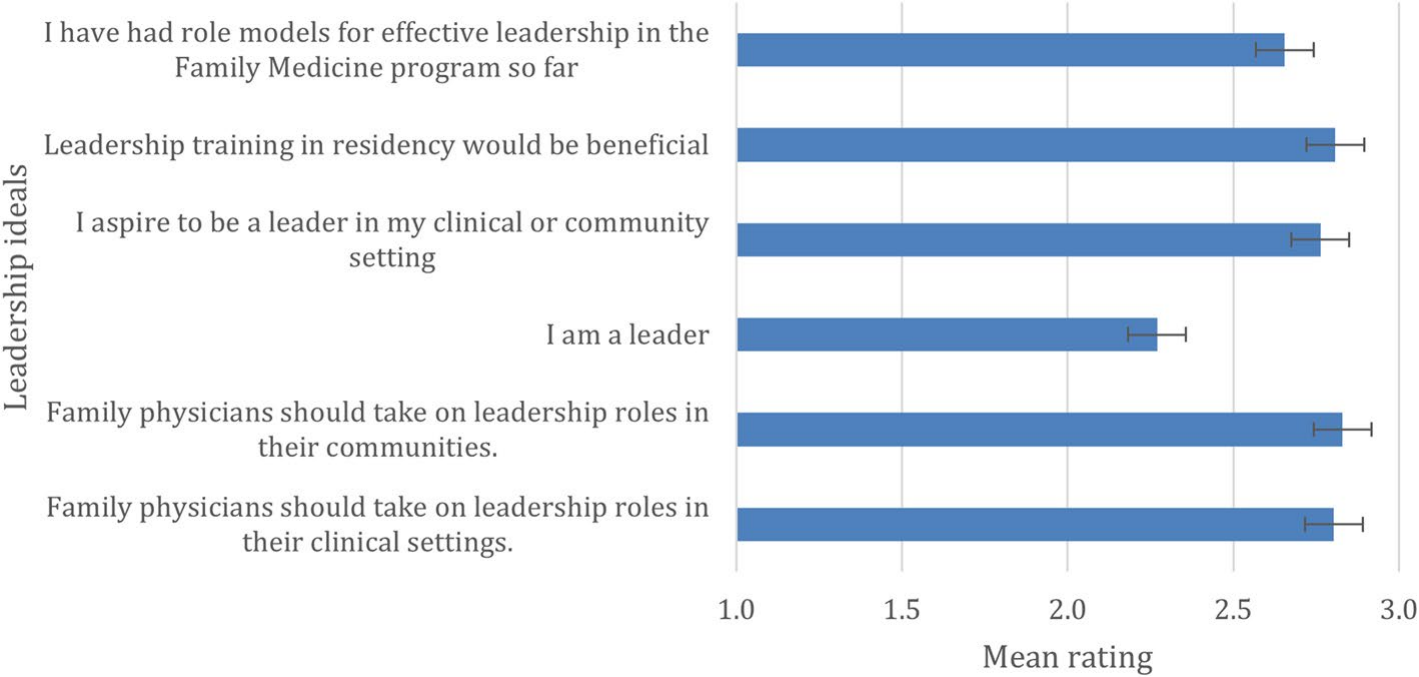
Mean rating of agreement with leadership ideals

In five out of six items regarding leadership ideals, affirmative answers were the most frequent, ranging from 70% of the agreement for 'I have had role models for effective leadership in the FM program' so far to 84.4% for 'Family physicians should take on leadership roles in their communities.' The only exception was a statement asking whether participants perceive themselves as leaders. In this case, most of the residents were neutral (62.6%); only 32.2% of the participants agreed, and 5.2% disagreed—the highest percentage for a negative answer regarding leadership ideals. Mean ratings of the agreement for each of the statements are in Fig. 1.

A paired-samples t-test was conducted to compare the mean ratings of the statements that were rated the highest and the lowest, respectively: 'Family physicians should take on leadership roles in their communities' (M = 2.83, SD = 0.41) and 'I am a leader' (M = 2.27, SD = 0.55). A significant difference was found, t(269) = 15.03, *p* < 0.001.

Subsequent analysis pertained to leadership domains. Table 1 presents percentages of residents, divided by residency year, who voiced a desire for more training in certain leadership domains (i.e., they chose 'None and desired more' or 'Some but not enough' as their answer).

**Table 1:** Percentages of residents desiring more training in certain leadership domains

**Leadership domain**	**Family Medicine Residents (%)**				Total (%)
	1^st^ Year	2^nd^ Year	3^rd^ Year	4^th^ Year	
Self-awareness	56.7%	62%	49.3%	60.3%	57%
Personal mastery	59.7%	70.4%	58%	58.7%	62%
Character development	52.2%	60.6%	60.9%	61.9%	59%
Professionalism	49.3%	52.1%	65.2%	61.9%	57%
Mentorship and coaching	64.2%	69%	59.4%	65.1%	64%
Self-management and balance	62.7%	62%	63.8%	57.1%	61%
Time management	65.7%	63.4%	65.2%	52.4%	62%
Lifelong learning	70.1%	56.3%	66.7%	63.5%	64%
Effective communication	61.2%	57.7%	63.8%	52.4%	59%
Social intelligence	58.2%	59.2%	60.9%	61.9%	60%
Cultural nuances	62.7%	63.4%	65.2%	61.9%	63%
Conflict resolution	68.7%	62%	71%	73%	69%
Feedback	67.2%	66.2%	69.6%	63.5%	67%
Relationships	59.7%	59.2%	68.1%	63.5%	63%
Development of others	59.7%	64.8%	69.6%	65.1%	65%
Teaching	68.7%	67.6%	62.3%	68.3%	67%
Effective teamwork	62.7%	64.8%	60.9%	57.1%	61%
Administration	58.2%	64.8%	73.9%	55.6%	63%
Ideals of a healthy workplace	56.7%	66.2%	69.6%	60.3%	63%
Collaboration and research	58.2%	66.2%	63.8%	63.5%	63%
Vision and goal setting	64.2%	66.2%	69.6%	57.1%	64%
Getting results	68.7%	62%	66.7%	60.3%	64%
Coalitions	62.7%	63.4%	71%	60.3%	64%
System transformation	67.2%	67.6%	72.5%	60.3%	67%

Overall, most of the residents (50% or more) who participated in the study voiced a desire for more training in all leadership domains.

Considering the highest percentages, the level of agreement between the leadership domains are conflict resolution (69%), teaching (67%), feedback (67%), and system transformation (67%).

On the other hand, self-awareness was one of the least frequently chosen domains for first-year (56.7%) and third-year (49.3%) residents. Moreover, first-year (49.3%) and second-year (52.1%) participants expressed low demand for training in professionalism. Finally, effective communication training was desired by 57.7% of second-year and 52.4% of fourth-year respondents.

**Fig. 2:**
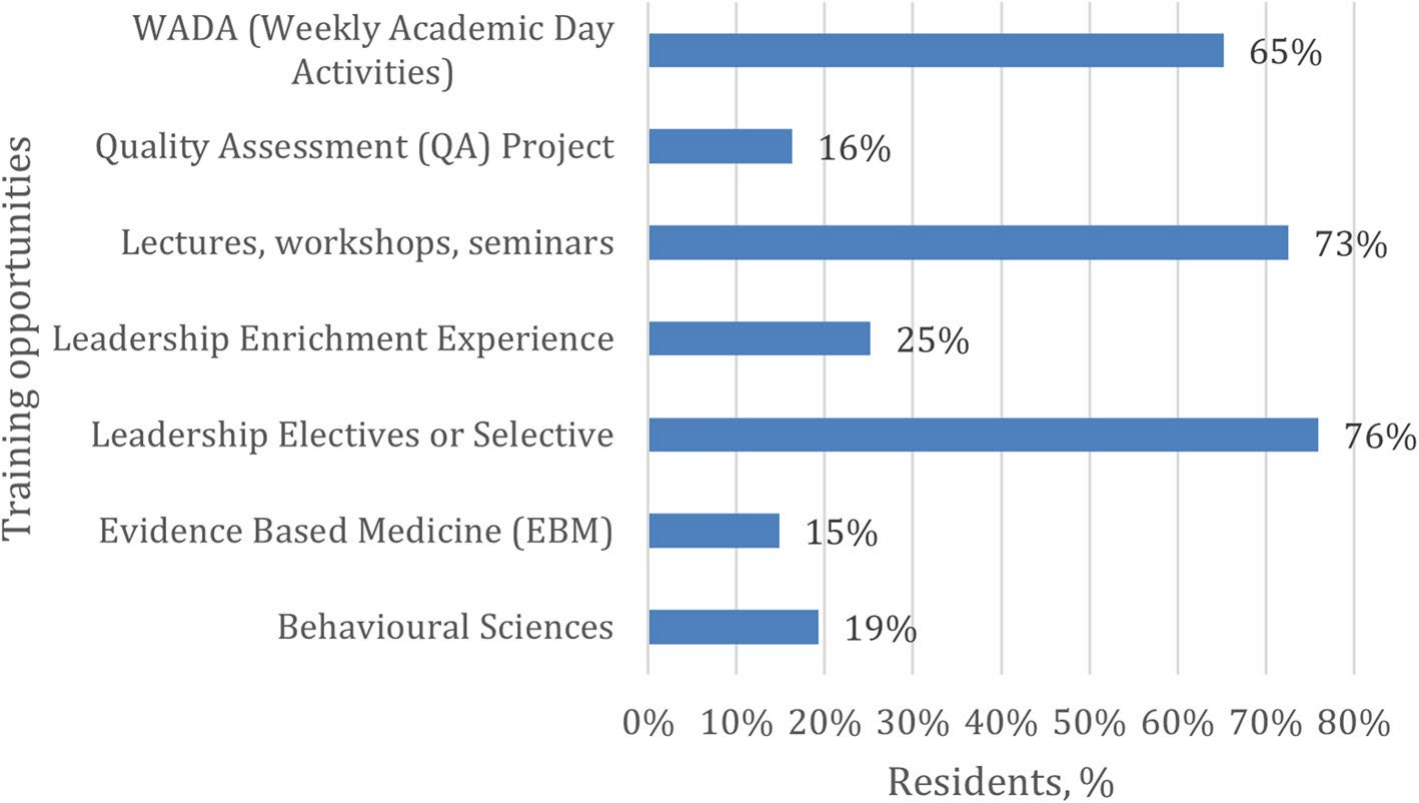
Training opportunities by percentages of residents

**Fig. 3:**
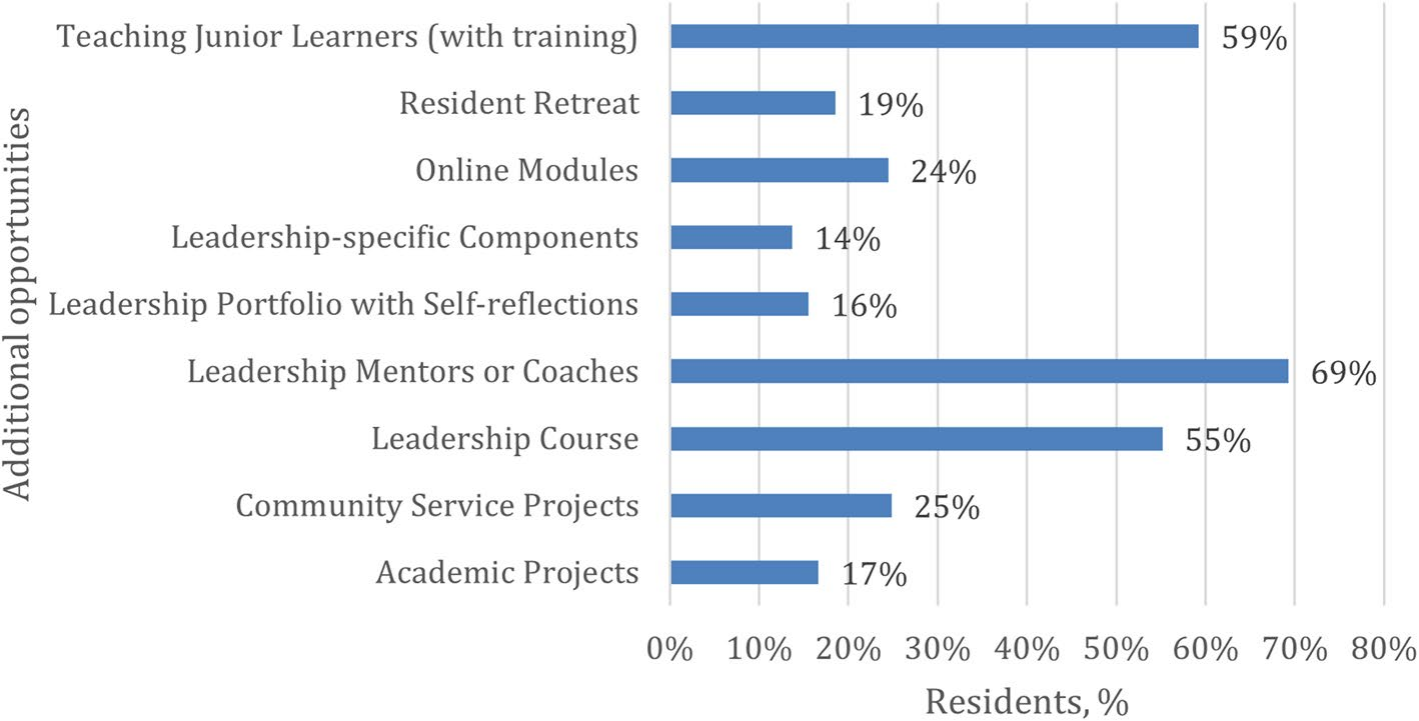
Additional opportunities by percentages of residents

Leadership opportunities were investigated next. As shown in Figs. 2 and 3, over 50% of residents indicated that leadership electives or selected lectures, workshops, or seminars, as well as WADAs (Weekly Academic Day Activities), leadership mentors or coaches, teaching junior learners (with training), and leadership courses could be incorporated into the curriculum to foster leadership skills. Finally, less than 25% of participants referred to behavioral sciences, quality assessment projects or evidence-based medicine, online modules, resident retreats, academic projects, leadership portfolios, and leadership-specific components (with clinical and other evaluations).

## Discussion

In our study, we found that FM residents associate family physicians with leadership, desire more personal and system-level leadership training, and think that leadership training may be increased in the current curriculum and established in new areas.

The significant difference (*p* < 0.001) between the Likert scale scores for the highest-ranked leadership ideal ('Family physicians should take on leadership roles in their clinical settings') and the lowest-ranked ('I am a leader') implies that there is room for growth regarding residents' development as leaders. The results are generally higher than those of other studies that used the same scale [5, 7].

In resident education, current leadership curriculum guidelines emphasize the development of lower-level leadership skills and knowledge [3]. The current curriculum has undoubtedly been significantly developed from the previous curriculum, but according to the study results, trainees still desire to learn more comprehensive leadership skills [4]. These results show that trainees' need to improve their leadership skills was greater than in the previous study in Canada [5] and similar previous studies conducted in many countries. This confirms trainees' need for more training in several leadership skills, giving the impression that a curriculum focusing more on leadership skills must be developed [7–9]. 

Conflict resolution (69%), teaching (67%), feedback, and system transformation (67%) are among the more advanced concepts that residents want to learn more about, which was higher than the need for training in Canada [5]. To account for statistical differences across domains, there was no curriculum focused specifically on leadership available at the time of this study.

There was a preference for both experiential and didactic learning opportunities. Curriculum components requiring some deliverable documents, such as leadership portfolios, were considered less desirable. This may be due to the nature and complexity of the current portfolio, which caused the trainees not to prefer it as a means of developing their leadership skills. This calls for studies to evaluate its effectiveness and ways to develop it, considering trainees' experience, opinion of development, and satisfaction.

Residents' level of agreement with several leadership ideals was unaffected by age, gender, or year of training. The desire for more training among third- and fourth-year residents is comparable to that of first- and second-year residents, which could be explained by a lack of substantial exposure to leadership domain training throughout their residency. Regarding leadership ideals, little variation existed between first- and fourth-year residents. A need may exist to provide residents with proper leadership training and competency, as there was no formal leadership training available when this poll was conducted.

Most residents (76%) indicated that leadership electives could be incorporated into the curriculum to foster leadership skills, which was higher than the percentage in Canada [5]. This may also be an opportunity to conduct studies to determine the importance of adding some new electives or rotations to the curriculum, the extent of the feasibility of some of the existing rotations and evaluate the possibility of modifying the rotations to be less lengthy and more numerous, especially after reducing the years of training in the new curriculum to three years.

Also, most residents (65%) indicated that WADAs are a part of developing their leadership skills and a good indicator for confirming the trainees' belief in the importance of this day in developing their academic skills, allowing them to evaluate the possibility of developing this day to include other skills, including leadership skills. Teaching junior learners (with training) has been suggested by most of the trainees (59%) to develop their skills, which may be a chance to consider adopting it as part of the curriculum. Overall, the percentage of residents desiring more leadership training in Saudi Arabia is higher than in Canada in all leadership domains [5].

### Strengths and limitations

A quantitative cross-sectional survey is the best way to get a wide range of views. In addition, only a modest number of people answered the survey. Only 30% of Riyadh's 900 residents responded to the questionnaire, which was sent to all the training centers in the city. Furthermore, due to the quantitative nature of this study, we were only able to collect a limited amount of information. One of the most pressing needs is to learn how residents view leadership and whether or not they believe family physicians should adopt a particular leadership style. Finally, there are no other local or regional studies to compare our findings to.

## Next step

There may be an opportunity to repeat this study from time to time to assess the extent of resident doctors' satisfaction with the development of the leadership curriculum. In addition to the possibility of applying the same study to programs of other specialties. There are also opportunities to conduct these same studies in all Kingdom programs.

## Conclusion

Residents were enthusiastic about the idea of family physicians being leaders, which aligned with the current educational philosophy of requiring formal training. They also indicated areas where leadership training might be improved and developed in the current curriculum. The results of this poll could be used to help residents build leadership skills by incorporating them into a formal leadership curriculum.

## Supplementary Information

**Additional file 1.**

## Data Availability

The datasets used and/or analyzed during the current study are available from the corresponding author on reasonable request.

## Abbreviations

FM: Family Medicine
WADAs: Weekly Academic Day Activities
